# Destructive Aorto-Mitral Endocarditis: Two Valves for One Annulus

**DOI:** 10.21470/1678-9741-2020-0674

**Published:** 2023

**Authors:** Calixte de La Bourdonnaye Blossac, Ian Cummings, Amedeo Anselmi, Erwan Flecher

**Affiliations:** 1 Department of Thoracic and Cardiovascular Surgery, Pontchaillou Hospital, Rennes, France.

**Keywords:** Endocarditis, Aortic Valve, Mitral Valve, Surgery

## Abstract

Surgery for endocarditis of the aorto-mitral continuity can be a challenge in case of extensive tissue destruction. We report two cases of a modified monobloc reconstruction of the aortic and mitral valves and of the aorto-mitral fibrous body. Two valve bioprostheses were sutured to each other and implanted as a composite graft. A pericardial patch sutured to the valves was employed to reconstruct both the noncoronary sinus and the left atrial roof. This technical adjustment allows adaptation to variable anatomical conditions in these particularly difficult cases.

## INTRODUCTION

Infective endocarditis carries a particularly high morbidity and mortality in cases involving abscess and disruption of the aorto-mitral continuity^[1]^. Surgery requires debridement of all infected tissue and may require reconstruction of the aorto-mitral annulus and left atrial roof^[2]^. Previously described techniques for reconstruction include use of monobloc aorto-mitral homograft and of handmade aorto-mitral bioprosthetic or mechanical valve composite grafts^[3,4,5]^. As a novel possible adaptation of this technique to some cases marked by “extreme” tissue destruction, we describe a variant of the aorto-mitral monobloc valve implantation, adding a pericardial patch to reconstruct the noncoronary sinus, the left atrial roof, and the aorto-mitral continuity. All patients provided written consent prior to surgery for use of personal data to research purposes.

### Case Description

Case 1 was a 51-year-old patient with bivalvular (native mitral and bioprosthetic aortic) endocarditis. Transesophageal echocardiography revealed mitral and aortic vegetations (17 and 7 mm, respectively), a significant periprosthetic aortic leak, and central mitral regurgitation. An aortic root abscess extending towards the anterior mitral leaflet and the left atrial roof was also noted.

Case 2 was a 70-year-old patient with bivalvular (native mitral and aortic) endocarditis. Transesophageal echocardiography revealed a bicuspid aortic valve, severe aortic regurgitation, large mitral vegetation, severe mitral regurgitation, and a fistula between the left sinus of Valsalva and the left atrium.

Both cases had preserved ventricular function and normal coronary arteries.

## TECHNIQUE

Cardiopulmonary bypass (CPB) was established using bicaval cannulation and femoral reinjection. Cold Custodiol® cardioplegia was instilled. An oblique aortotomy was performed and the mitral valve approached via a transseptal incision extended towards the left atrial roof.

**Fig. 1:**
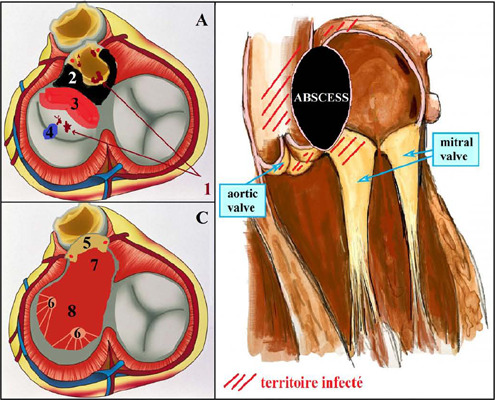
Infected tissues (A and B) and result after surgical excision (C). 1, vegetations; 2, abscess; 3, infected anterior mitral leaflet; 4, mitral perforation; 5, remaining aortic root after excision of all infected tissues - coronary ostia are preserved; 6, papillary muscles; 7, left outflow tract; 8, mitral orifice.

The infected aortic (bioprosthetic and native) valves, the native mitral valves, and part of ascending aorta had to be debrided. Anterior mitral leaflet infection, aorto-mitral disruption, and abscess involving two thirds of the aortic root were observed in both cases. The posterior leaflet of the mitral valve was free from infection (Figure 1). The sacrifice of the aorto-mitral fibrous continuity was required, leaving a single aorto-mitral neo-orifice. The coronary ostia and the aortic root (left and right coronary sinuses) were preserved.

**Fig. 2:**
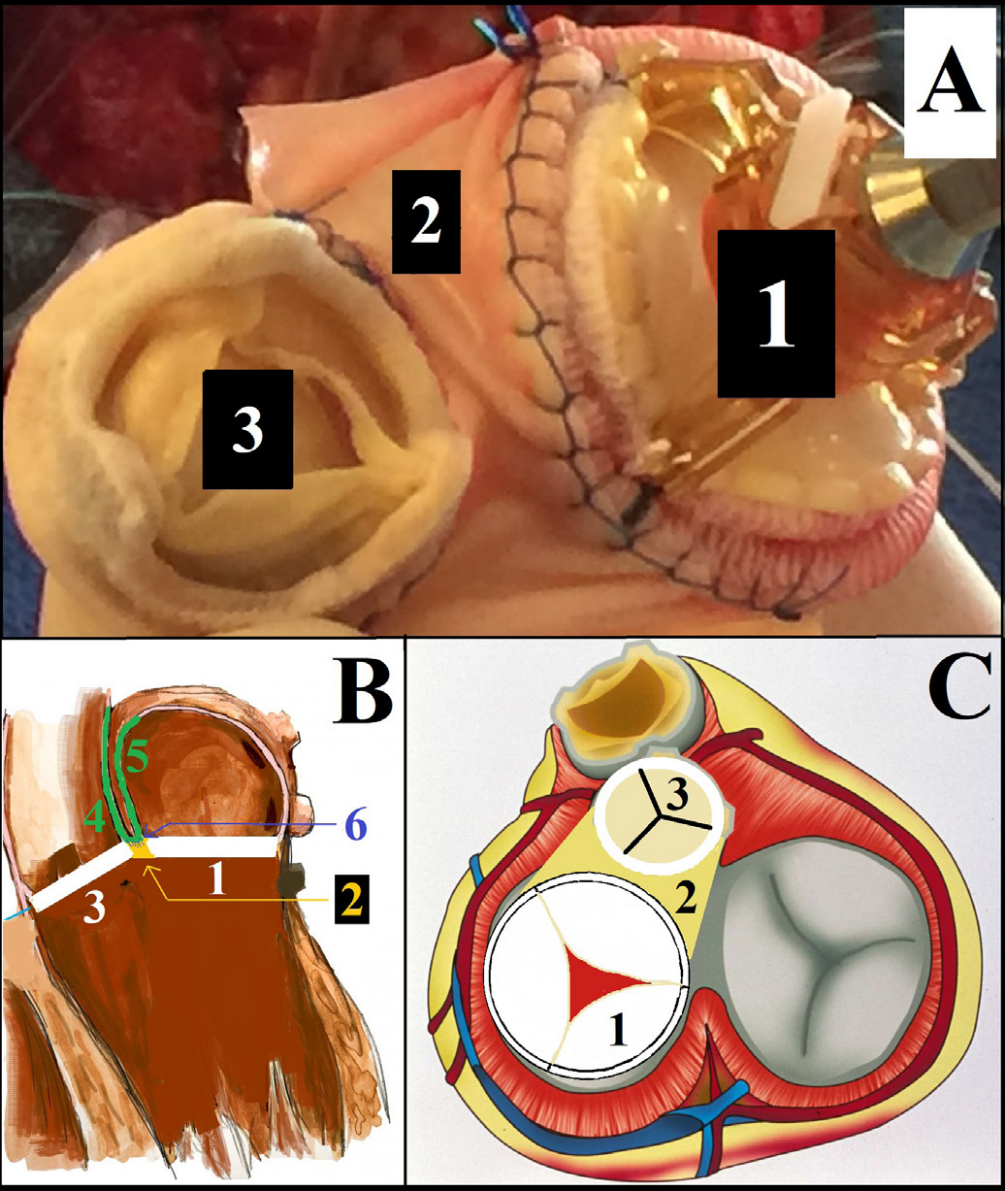
Picture of both valves sutured (A) and illustration of the repair (B and C). 1, mitral prosthesis; 2, pericardial patch between both valves; 3, aortic prosthesis; 4-5-6, second pericardial patch with a “V shape”; 4, reconstruction of the aortic wall; 5, reconstruction of the left atrial roof; 6, attachment of the V shape pericardial patch to the monobloc.

A modified “monobloc repair” was performed. Two Medtronic Mosaic© aortic valves (sized 25 and 23 mm, cases 1 and 2, respectively) were joined to two Abbott Epic© mitral valves (sized 29 and 31 mm, cases 1 and 2, respectively) (Figure 2), using a polypropylene 4/0 suture over approximately one third of their perimeter (an overlocking suture was used to maximize hemostasis). A 1.5-cm pericardial patch was inserted between the two valves in case 2 to allow more degree of freedom and adaptability of the composite graft; the patch was secured using again an overlocking suture. The bivalvular monobloc kit was sutured to the neo-orifice using interrupted pledgeted U-shaped stitches. The base of the U was placed on the left atrial side for the mitral portion and on the ventricular side for the aortic portion. In order to reconstruct both the aortic and left atrial walls, a second V-shaped pericardial patch was added: the bottom of the patch was sutured either to the aortic bioprosthesis (case 1) or to the pericardial patch portion inserted between the two prostheses (case 2); hence, the left side of the patch was used to reconstruct the ascending aorta and its right side to rebuild the left atrial roof. Aortic cross-clamping and CPB times were 229/284 minutes (case 1) and 192/237 minutes (case 2).

Intensive care unit stay was seven days in case 1 and 10 days in case 2. Both cases required permanent pacemaker implantation. Discharge echocardiography revealed satisfactory bivalvular function, as well as the control at one year of follow up. Both patients were discharged home with satisfactory functional status.

## DISCUSSION

The surgical management of infectious endocarditis is based on a radical excision of the infected tissues. Nonetheless, reconstruction can be difficult in some circumstances. Herein we describe a modified monobloc aorto-mitral replacement technique^[3,4]^, aimed at facilitating surgical reconstruction in cases with extensive destruction of the native aorto-mitral continuity, left atrial roof, and valve annuli due to infectious endocarditis.

The strategy presented here adjusts the previously described “monobloc” reconstruction techniques to cases with aortic root and coronary ostia preservation^[3,4]^. This modification entails the use of a V-shaped patch to reconstruct both the left atrium and the noncoronary aortic sinus; this patch is secured directly to the composite valve graft. When the destruction is limited to the noncoronary sinus at the level of the root, this strategy might help avoiding full root replacement and coronary buttons reimplantation^[3]^, and therefore reduces the complexity of the repair as well as the CPB and aortic cross-clamping times. Also, a distinct patch can be inserted between the two valves to provide versatility in their placement while respecting the native aortomitral angle. We also suggest performing valve sizing by inserting into the defect both the aortic and mitral valve sizers at the same time, in order to understand the prospected relationship of the two valves.

We underline the usefulness of bicaval canulation and a biatrial access to achieve an optimal exposure of the lesions (namely, left atrial roof and anterior mitral annulus) and facilitate complex reconstructions. We suggest that bicaval cannulation should be liberally employed in patients operated on for echocardiographically evident extensive abscess of the aortomitral body.

## CONCLUSION

This modified monobloc aorto-mitral valve implantation should be considered as part of the toolbox for surgical management of severe infectious endocarditis. It enhances the possibilities to adapt the reconstruction to variable anatomy defects after debridement.

## Abbreviations, Acronyms & Symbols

CPB: Cardiopulmonary bypass
